# The complete plastome of a folk medicinal herb *Isodon lophanthoides* var. *graciliflorus*

**DOI:** 10.1080/23802359.2020.1768949

**Published:** 2020-05-28

**Authors:** Hui-Ye Zhang, Jing Xia, Wen-Zhe Ma

**Affiliations:** a Hutchison Whampoa Guangzhou Baiyunshan Chinese Medicine Co., Ltd., Guangzhou, China; b Guangdong AIB Polytechnic College, Guangzhou, China

**Keywords:** Plastome, medicinal herb, phylogenetic tree, *Isodon lophanthoides* var. *graciliflorus*

## Abstract

*Isodon lophanthoides* var. *graciliflorus* is a folk medicinal herb that is distributed in tropical and subtropical Asia. In this study, the complete plastome of *I. lophanthoides* var. *graciliflorus* was assembled and annotated. The plastome is 152,195 bp in length, consisting of a large single-copy (LSC) region of 83,095 bp, a small single-copy (SSC) region of 17,699 bp, and two inverted repeat (IR) regions of 25,701 bp, each. It has 113 genes, including 80 protein-coding genes, 29 tRNA genes, and 4 rRNA genes. The overall GC content of the plastome is 37.6%. Phylogenetic analysis showed that *I. lophanthoides* var. *graciliflorus* was sister to *Isodon serra*.

*Isodon lophanthoides* var. *graciliflorus* (Benth.) H. Hara, which is distributed in China, India, Laos, Myanmar, Nepal, Thailand and Vietnam, belongs to the genus *Isodon* in the family Lamiaceae (Wu and Li 1977; Li and Hedge 1994). *Isodon lophanthoides* var. *graciliflorus*, together with *Isodon lophanthoides* var. *lophanthoides* and *Isodon serra* (Maximowicz) Kudô, has been considered as botanical source of the traditional medicine *Xihuangcao*, which is commonly used in South China as a *dampness*-draining, anicteric and liver protection herb (Guangdong Food and Drug Administration 2011). However, most of the *Xihuangcao*-based products do not specify the taxon used. Additionally, due to the different secondary metabolites of these taxa, it is controversial whether they can be used equally (Wong et al. 2016). In this study, we characterized the complete plastome of *I. lophanthoides* var. *graciliflorus* to better survey the resources of *Xihuangcao* in the future.

The plant material of *I. lophanthoides* var. *graciliflorus* was sampled from Hutchison Whampoa Guangzhou Baiyunshan Chinese Medicine Co., Ltd (N23°11′11′′, E113°15′57′′). The voucher specimen (Zhy-Z2) was deposited at South China Botanical Garden Herbarium. Total genome DNA was extracted from fresh leaves using a modified CTAB protocol (Doyle and Doyle 1987). Library construction and paired-end sequencing were performed by the Beijing Genomics Institute (Wuhan, China). Genome sequences were assembled in SPAdes v3.10.1 (Bankevich et al. 2012); and Geneious Prime 2019 (Biomatters, Ltd., Auckland, New Zealand) was subsequently used to close gaps. The genes in the plastome were annotated and manually adjusted using Geneious Prime 2019. Phylogenomic analysis was performed using the complete plastomes from 24 taxa in the Lamiaceae (including *I. lophanthoides* var. *graciliflorus* in this study). The plastome sequences were aligned using MAFFT (Katoh and Standley 2013). The phylogenetic tree was constructed with RAxML (Stamatakis 2014), using the maximum-likelihood algorithm.

The *I. lophanthoides* var. *graciliflorus* plastome (GenBank accession no.: MT317098) is 152,195 bp in length, consisting of a large single-copy (LSC) region of 83,095 bp, a small single-copy (SSC) region of 17,699 bp, and two inverted repeat (IR) regions of 25,701 bp, each. The plastome of *I. lophanthoides* var. *graciliflorus* contains 113 genes, including 80 protein-coding genes, 29 tRNA genes, and 4 rRNA genes. The overall GC content is 37.6%, higher than those of LSC (35.6%) and SSC (31.0%) regions, but lower than those of IR regions (43.1%). Based on the ML tree, all sampled members of tribe Ocimeae formed a clade, and *I. lophanthoides* var. *graciliflorus* was sister to *I. serra* (Figure 1). The plastome of *I. lophanthoides* var. *graciliflorus* is of significance for its evolutionary studies and medicinal researches.

**Figure 1.**
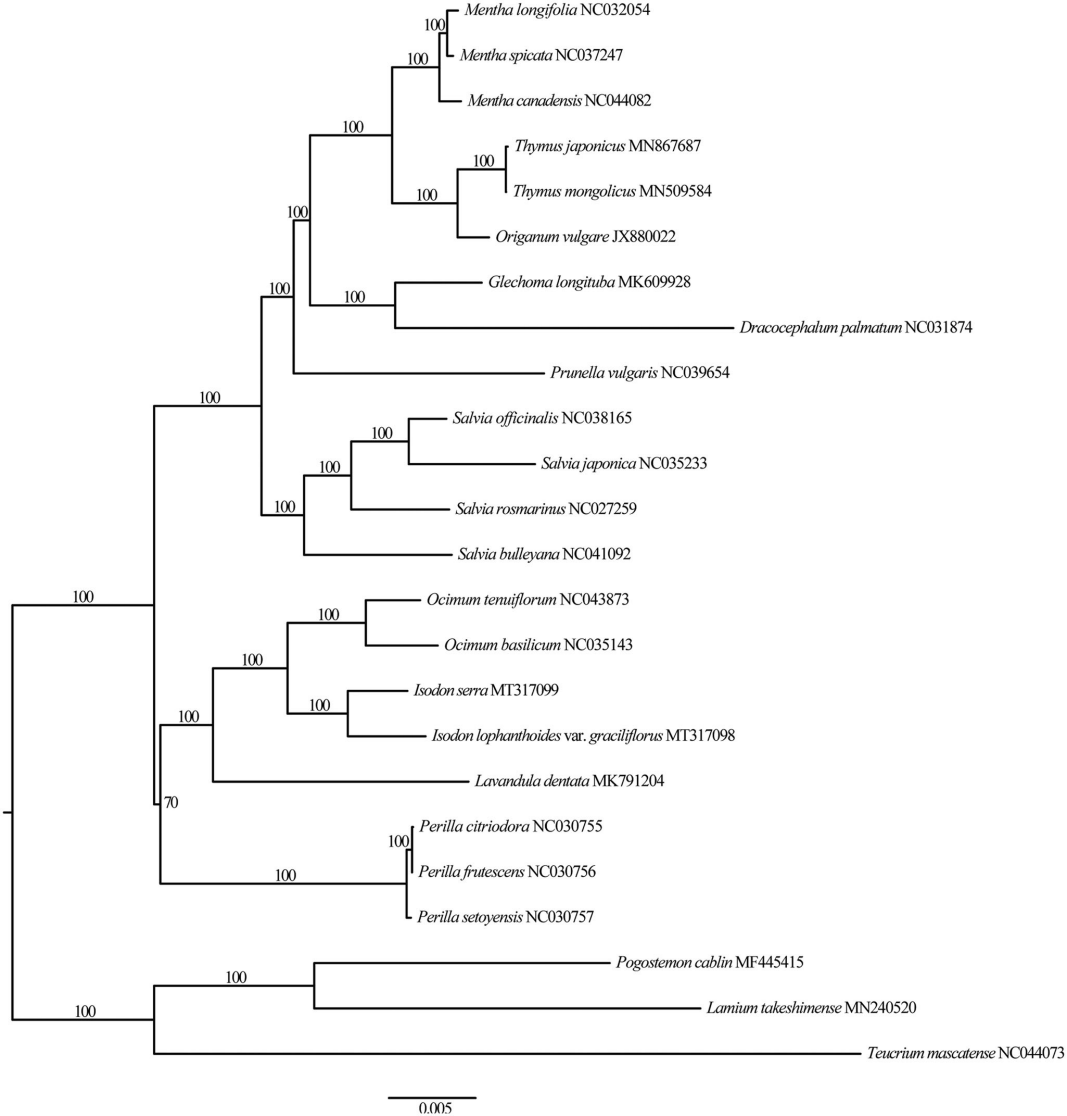
Maximum-likelihood tree inferred from chloroplast genome sequences of 24 taxa. The bootstrap values were based on 1000 replicates. The scale for nucleotide substitutions is showed in legend below the tree.

## Data Availability

The data that support the findings of this study are openly available in GenBank at https://www.ncbi.nlm.nih.gov/, reference number MT317098.

## References

[CIT0001] Bankevich A, Nurk S, Antipov D, Gurevich AA, Dvorkin M, Kulikov AS, Lesin VM, Nikolenko SI, Pham S, Prjibelski AD, et al. 2012. SPAdes: a new genome assembly algorithm and its applications to single-cell sequencing. J Comput Biol. 19(5):455–477. 22506599 10.1089/cmb.2012.0021 PMC3342519

[CIT0002] Doyle JJ, Doyle JL. 1987. A rapid DNA isolation procedure for small quantities of fresh leaf tissue. Phytochem Bull. 19:11–15.

[CIT0003] Guangdong Food and Drug Administration. 2011. Guangdong Chinese Materia Medica Standards. Guangdong (China): Guangdong Science & Technology Press; p. 347–353.

[CIT0004] Katoh K, Standley DM. 2013. MAFFT multiple sequence alignment software version 7: improvements in performance and usability. Mol Biol Evol. 30(4):772–780. 23329690 10.1093/molbev/mst010 PMC3603318

[CIT0005] Li XW, Hedge IC. 1994. Lamiaceae. In Flora of China. Beijing (China): Science Press; St. Louis (MO): and Missouri Botanical Garden; p. 50–299.

[CIT0006] Stamatakis A. 2014. RAxML version 8: a tool for phylogenetic analysis and post-analysis of large phylogenies. Bioinformatics. 30(9):1312–1313. 24451623 10.1093/bioinformatics/btu033 PMC3998144

[CIT0007] Wong LL, Liang ZT, Chen HB, Zhao ZZ. 2016. Rapid differentiation of Xihuangcao from the three *Isodon* species by UPLC-ESI-QTOF-MS/MS and chemometrics analysis. Chin Med. 11:48. 28018478 10.1186/s13020-016-0120-y PMC5160003

[CIT0008] Wu CY, Li HW. 1977. Flora Reipublicae Popularis Sinicae. Beijing (China): Science Press, 66: p. 482.

